# The evolution of indirect reciprocity under action and assessment generosity

**DOI:** 10.1038/s41598-021-96932-1

**Published:** 2021-08-31

**Authors:** Laura Schmid, Pouya Shati, Christian Hilbe, Krishnendu Chatterjee

**Affiliations:** 1 IST Austria, Am Campus 1, 3400 Klosterneuburg, Austria; 2 Department of Computer Science, University of Toronto, Toronto, ON M5S Canada; 3 Max Planck Research Group Dynamics of Social Behavior, Max Planck Institute for Evolutionary Biology, 24306 Ploen, Germany

**Keywords:** Evolutionary theory, Social evolution

## Abstract

Indirect reciprocity is a mechanism for the evolution of cooperation based on social norms. This mechanism requires that individuals in a population observe and judge each other’s behaviors. Individuals with a good reputation are more likely to receive help from others. Previous work suggests that indirect reciprocity is only effective when all relevant information is reliable and publicly available. Otherwise, individuals may disagree on how to assess others, even if they all apply the same social norm. Such disagreements can lead to a breakdown of cooperation. Here we explore whether the predominantly studied ‘leading eight’ social norms of indirect reciprocity can be made more robust by equipping them with an element of generosity. To this end, we distinguish between two kinds of generosity. According to assessment generosity, individuals occasionally assign a good reputation to group members who would usually be regarded as bad. According to action generosity, individuals occasionally cooperate with group members with whom they would usually defect. Using individual-based simulations, we show that the two kinds of generosity have a very different effect on the resulting reputation dynamics. Assessment generosity tends to add to the overall noise and allows defectors to invade. In contrast, a limited amount of action generosity can be beneficial in a few cases. However, even when action generosity is beneficial, the respective simulations do not result in full cooperation. Our results suggest that while generosity can favor cooperation when individuals use the most simple strategies of reciprocity, it is disadvantageous when individuals use more complex social norms.

## Introduction

People tend to put in time and effort to achieve and maintain a good standing with those that surround them^1–4^. This human concern for a good reputation can be an important motivation to engage in cooperative behavior: Once somebody’s reputation is at stake, people have more of an incentive to engage in costly acts of helping others^5–10^. This reputation-based mechanism for the evolution of cooperation is referred to as indirect reciprocity^11–14^. In contrast to direct reciprocity^15–18^, which is based on repeated interactions between the same players, indirect reciprocity does not require any two individuals to interact with one another more than once. Instead it only requires population members to continually assess each others’ actions, and to act based on these reputations. Whereas direct reciprocity is based on individual memories of interactions with a given group member, indirect reciprocity builds upon a group’s collective memories.

In order to analyze the process by which reputations evolve, the literature on indirect reciprocity considers certain social dilemma situations^11–14^. In the simplest case, each dilemma situation involves only two population members, who are referred to as ‘donor’ and ‘recipient’, respectively. The donor is asked whether or not to pay a personal cost to provide help to the recipient. The two possible actions of either offering help or refusing help are interpreted as cooperation and defection, respectively. While the recipient does not make any decisions, the donor’s action is observed by other population members. These observers then decide how the donor’s reputation should be updated in light of their behavior.

How observers update reputations, and how donors decide whom to help, depends on the social norm applied in the population. These social norms consist of two components. First, the social norm’s assessment rule specifies which behaviors of the donor should improve the donor’s reputation, and which behaviors should be condemned. Second, the social norm’s action rule tells the donor whether or not to help the recipient; this decision may in turn depend on the recipient’s and the donor’s current reputation. A well-known example of a social norm is ‘Image Scoring’^19–21^. According to the assessment rule of image scoring, a donor’s reputation should improve every time the donor cooperates, and it should deteriorate every time the donor defects. According to its action rule, a donor should only cooperate with those recipients who are sufficiently well-reputed. Importantly, the social norm of Image Scoring has the property that assessments only depend on whether or not the donor cooperated. Social norms with this property are sometimes referred to as ‘first-order’ norms. While this basic principle of first-order norms may appear intuitive, Image Scoring has been shown to be unstable^22–24^. The reason for this instability is that individuals are required to defect against an ill-reputed group member; yet by doing so, they harm their own reputation. To overcome this inconsistency, it has been argued that stable norms of indirect reciprocity need to be sufficiently complex to differentiate between justified and unjustified acts of defection^22,23^.

To identify such stable cooperative norms, the landmark papers by Ohtsuki and Iwasa^25,26^ consider an even simpler decision situation. In their setup, reputations are required to be binary, such that individuals can either be ‘good’ or ‘bad’. For this binary model of reputations, they allow for up to all ‘third-order’ assessment rules. In addition to the donor’s action, third-order assessments may also depend on the current reputation of the donor and the recipient. For example, while helping a good recipient may be assessed as good, the very same action towards a bad recipient may be assessed as bad. With an exhaustive search on the space of all third-order norms, Ohtsuki and Iwasa identified eight successful norms that can maintain cooperation, which they coined the “leading eight”. All leading eight norms agree that a cooperative action towards a good recipient should yield a good reputation, whereas a defecting action towards a good recipient should yield a bad reputation. The norms differ, however, in how they evaluate interactions with a bad recipient. While some norms find it acceptable if help is provided to a bad recipient, other norms like ‘Stern Judging’^27^ condemn such behaviors completely.

The findings of Ohtsuki and Iwasa have been hugely influential for the further development of the field^27–32^, yet they are based on a number of important assumptions. One of their key assumptions is that reputations are assigned publicly. This means that after any interaction between a donor and recipient, it is a central authority that assigns an updated reputation to the donor; this publicly assigned reputation is then adopted by the entire population. As a consequence, the views of different individuals are always perfectly correlated: if a given group member is considered to be good by one individual, they are also considered to be good by anyone else. The assumption of public information greatly facilitates the mathematical analysis of indirect reciprocity. However, it cannot capture scenarios in which individuals make their own judgments, based on their own private information. When individuals make their own judgments, they may start to disagree about which reputation they assign to a given group member. Such disagreements can arise, for example, when individuals differ in which interactions they observe, or when they occasionally misinterpret a given interaction. Once such disagreements arise, they can further proliferate, because individuals now also perceive all future interactions of that group member differently^33–35^. For private, scarce, and incomplete information, individual-based simulations thus suggest that the leading-eight social norms no longer effectively promote the evolution of cooperation^36^.

This finding naturally calls for mechanisms to mitigate the effect of noisy environments and private information on indirect reciprocity^37,38^. Here we explore the effect of a particular mechanism, generosity. The value of generosity has previously been stressed in the literature on direct reciprocity^39–43^. For example, by occasionally cooperating against defectors, the strategy ‘Generous Tit-for-Tat’ can stabilize full cooperation^39,40^, even though the classical Tit-for-Tat strategy cannot. Similarly, the analogous first-order social norm ‘Generous Scoring’ has been shown to be stable in the context of indirect reciprocity^44^, even though classical Image Scoring is not. In both cases, there is the same intuition for why a certain degree of generosity is favorable. By becoming more generous, populations of reciprocators are more likely to sustain cooperation among themselves even in noisy environments. This in turn makes them more robust against invasion by unconditional cooperators. At the same time, however, reciprocators must not be too forgiving, for otherwise they can be invaded by unconditional defectors. The optimal degree of generosity thus needs to strike a balance between being sufficiently forgiving to correct errors, and being sufficiently strict to avoid exploitation.

In the following, we propose a framework to incorporate an element of generosity into the leading eight social norms. To this end, we distinguish between two kinds of generosity. The first, assessment generosity, makes individuals more generous when assigning reputations to other group members. In situations in which they would usually assign a bad reputation to a given group member, they may occasionally assign a good reputation instead. The second, action generosity, makes individuals more generous in their decisions whom to help as a donor. In situations in which they would usually defect, they may occasionally cooperate instead. Although these two kinds of generosity may seem similar, our results suggest that their effect on the resulting reputation dynamics is very different. In particular, while action generosity can sometimes enhance cooperation, assessment generosity is always detrimental. Moreover, even under action generosity, we do not observe the evolution of high cooperation rates when environments are noisy. Our results suggest that complex social norms of indirect reciprocity are not compatible with individual acts of generosity. Unless generosity is the result of a coordinated effort among all population members, generosity merely acts as another seed of disagreement.

## Results

### A theoretical framework of action and assessment generosity

To incorporate generosity into higher-order social norms of indirect reciprocity, we consider a well-mixed population of fixed size *N*. In every round, two players are randomly chosen to engage in an interaction. One of the players is randomly assigned to be the donor, whereas the other player is assigned to be the recipient. The donor chooses whether to confer a benefit *b* to the recipient, at an own cost of *c*, with $0 \!<\! c \!<\! b$. Other members of the population independently observe the donor’s decision with probability *q*. We assume that information is private: every observer keeps track of others’ reputations by updating their personal reputation repository, and there is no shared judgment of observed actions (see “Methods”). Players thus individually update and keep track of their opinion about others based on their observations. These observations are subject to noise: observers can misperceive an action with a probability $\varepsilon$, and mistakenly interpret e.g. cooperation as defection.

**Figure 1:**
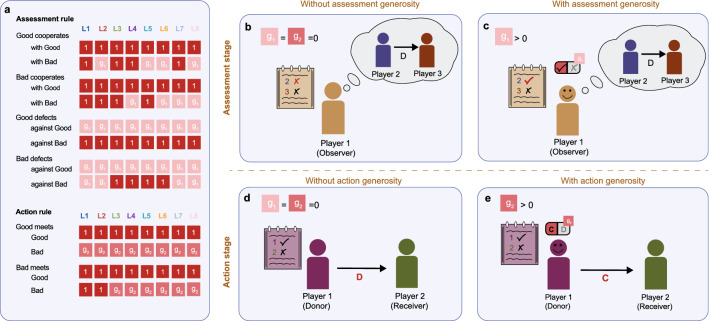
The leading eight social norms with assessment and action generosity. (**a**) To sustain cooperation based on indirect reciprocity, Ohtsuki and Iwasa^26^ suggested a set of eight social norms. Each social norm consists of an assessment rule and an action rule. The assessment rule determines with which probability observers assign a good reputation to a given donor. This assessment depends on the donor’s action (cooperation or defection) and on the reputations of the donor and the recipient (good or bad). The action rule determines with which probability a donor cooperates with a given recipient. Again, this choice depends on the reputation of the donor and the recipient. The original leading eight social norms are deterministic, such that all probabilities are either zero or one. In our framework, we introduce stochasticity by allowing individuals to be generous. We distinguish two kinds of generosity. (**b**,**c**) Assessment generosity means that every time individuals usually assign a bad reputation, they instead assign a good reputation with probability $g_1$. (**d**,**e**) Action generosity means that every time individuals usually defect, they instead cooperate with probability $g_2$.

How observers update others’ reputation based on what they observe, and how they subsequently act towards others, is governed by their social norm. We broadly interpret social norms as rules that tell individuals how they should behave in social interactions. In our context, we consider norms that consist of two components: an assessment rule and an action rule^14^. The assessment rule prescribes how reputations are updated, based on the observed actions and the possible context of an interaction. The action rule governs a player’s behavior when it is their turn to decide whether to confer a benefit to a co-player. Following Ohtsuki and Iwasa^25,26^, we assume that reputations are binary (good or bad), and social norms are at most third order. To allow for generosity, we consider modified versions of the deterministic leading eight social norms *L*1–*L*8 (see Fig. 1a). Players with these modified versions always cooperate if the original leading eight prescribe to do so. Similarly, they always assign a good reputation in cases where the original version would. However, in cases where the original social norm would assign a bad reputation, the modified version instead assigns a good reputation with probability $g_1$ (Fig. 1b,c). Analogously, in cases where the original leading eight norm prescribes defection, the modified version instead cooperates with probability $g_2$ (Fig. 1d,e). We refer to $g_1$ and $g_2$ as the probabilities that players engage in assessment generosity and action generosity, respectively. In the limiting case with $g_1\!=\!g_2\!=\!0$, we recover the original leading eight.

We note that our framework makes two assumptions about the stochasticity we introduce. For one, players are only forgiving, but not spiteful: players never defect when the original leading eight rule prescribes cooperation, and they never assign a bad reputation to a player they are supposed to regard as good. This means that only when the original norm prescribes a negative assessment or defection, there is a probability to positively assess ($g_1$) or cooperate ($g_2$) instead. Another simplifying assumption is that players use the same probability $g_1$ for all instances in which assessment generosity can be applied (similarly, they use the same probability $g_2$ for all instances in which action generosity can be applied). For example, an observer with social norm Stern Judging (or *L*6) makes no distinction between a player who cooperates with a bad recipient and a player who defects with a good recipient. In both cases, the observer generously assigns a good reputation with probability $g_1$.

### Reputation dynamics under assessment generosity

In the following, we first explore each kind of generosity in isolation. We start by considering the effects of assessment generosity. To this end, we first take the players’ norms as given. Different players may adopt different norms, but each player’s social norm is fixed in time. We can then describe how the players’ reputations change over time with a so-called image matrix^33^ $M(t)\!=\!\big (m_{ij}(t)\big )$. This image matrix represents a collection of the players’ reputation repositories. An entry $m_{ij}(t)\!=\!1$ indicates that player *i* assigns a good reputation to *j* at time *t*. Similarly, an entry $m_{ij}(t)\!=\!0$ indicates that *i* regards *j* as bad. After every round of the game, *M*(*t*) is updated. The updated image matrix depends on which players are chosen to be the donor and the recipient, on the donor’s action, on who observes the interaction, and on the social norms applied by the observers. For example, if an individual *j* is regarded as good by all players, but *j* defects against some other good individual, then some of the entries of the image matrix may change from $m_{ij}(t)\!=\!1$ to $m_{ij}(t\!+\!1)\!=\!0$.

**Figure 2:**
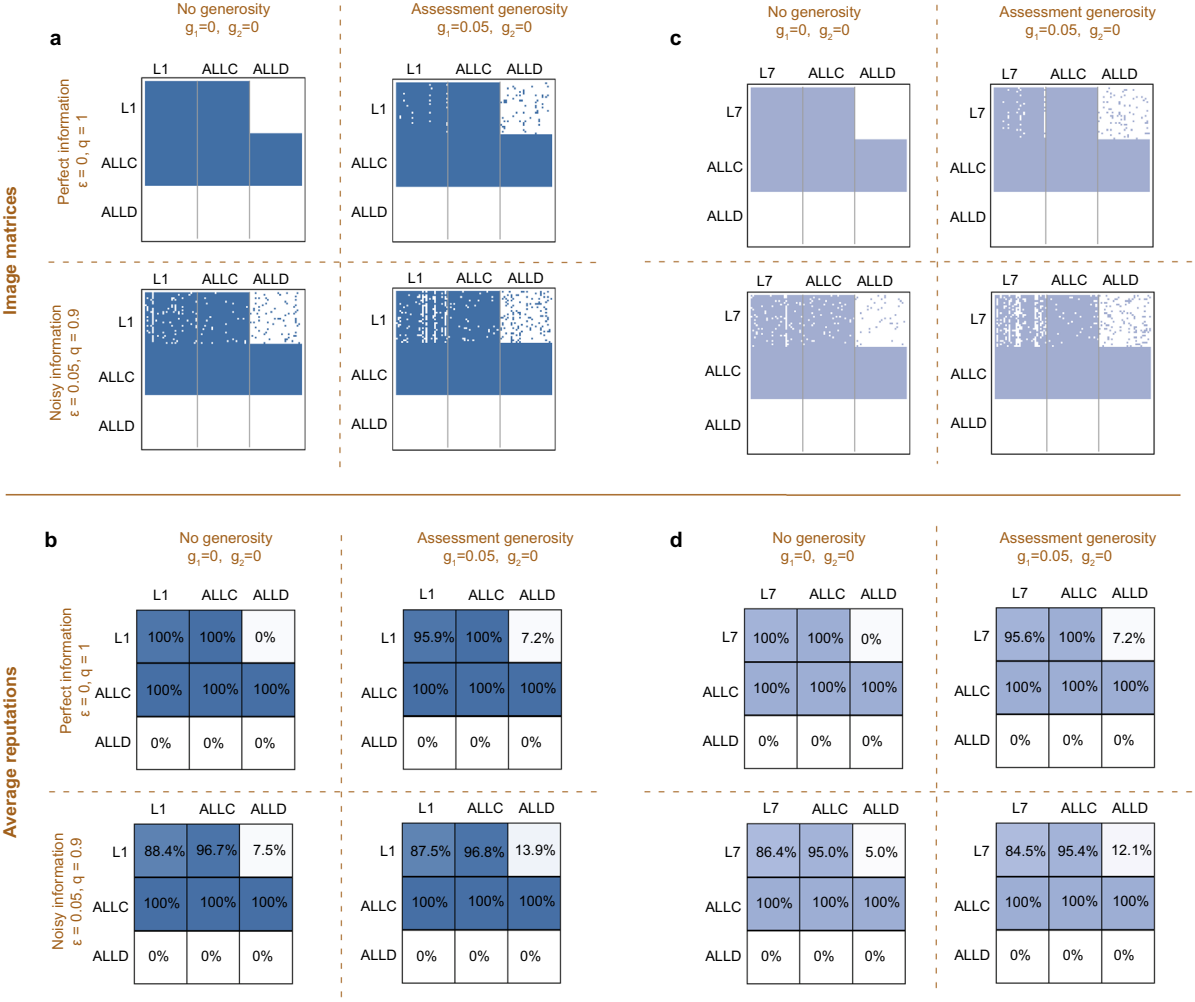
The effect of assessment generosity on the dynamics of reputations. (**a**) Image matrices are representations of how players assess each other at any given time. To depict these image matrices graphically, a colored dot means that the corresponding row player attributes a good image to the corresponding column player. Here, we show snapshots of such image matrices when players either use the leading-eight social norm $L_1$, *ALLC*, or *ALLD* (in equal proportions). We consider four scenarios. These scenarios differ in whether information is perfect or noisy, and in whether or not $L_1$ players are generous. When information is perfect and there is no generosity, we observe that the reputation assignments of different $L_1$ players are perfectly correlated. If one $L_1$ player assigns a good reputation to some other group member, then so does every other $L_1$ player. In contrast, the presence of either noise or generosity introduces disagreements among $L_1$ players. (**b**) Here, we show the average image players have of one another. Generosity makes $L_1$ players perceive each other less favorably, and it makes them perceive *ALLD* players more favorably, irrespective of whether information is perfect or noisy. (**c**,**d**) We observe similar patterns for all other leading eight social norms. Here we illustrate the competition between $L_7$, *ALLC*, and *ALLD*. Parameters: We use a population of size $N\!=\!90$, an error rate of either $\varepsilon \!=\!0$ or $\varepsilon \!=\!0.05$, and a generosity probability of either $g_1\!=\!0$ or $g_1\!=\!0.05$. Simulations are run for $2\cdot 10^6$ iterations, and the initial image matrix assumes a good reputation for all players.

Let us illustrate the concept of image matrices with an example. To this end, we consider a population that in equal proportions applies one of the social norms $L_1$, *ALLC*, *ALLD*. Here, *ALLC* is the (trivial) social norm that assigns a good reputation to all behaviors, and that prescribes to cooperate with everyone. Similarly, *ALLD* is the norm that uniformly assigns bad reputations and that always prescribes to defect. We consider four scenarios, depending on whether or not information is noisy, and depending on whether or not $L_1$ displays some assessment generosity (Fig. 2a). In the baseline case of no noise and no generosity, we observe that $L_1$ perfectly distinguishes between different players. Eventually, all $L_1$ players assign a good reputation to each other and to all unconditional cooperators, whereas they assign a bad reputation to all defectors. Once we allow for perception errors, however, there is no longer a perfect match between the players’ norms and their reputations. For instance, there is now a 7.5% chance that $L_1$ players regard an *ALLD* opponent as good despite their constant defection. Similarly, $L_1$ players now consider each other as bad with an average probability of $11.6\%$ (Fig. 2b). Given that the error rate is only 5%, this increase in bad assessments cannot be explained by the direct effect of errors alone. Instead, the original disagreements caused by errors trigger further disagreements over time^36^. It is exactly this excess in bad assessments that generosity might help to reduce.

Surprisingly, we find that assessment generosity rather has the opposite effect. It further increases the mismatch between the players’ social norms and their reputations (Fig. 2a, right panels). Even in the absence of errors, generosity increase the likelihood that $L_1$ players regard each other as bad (from 0 to 4.1%). Similarly, it increases the likelihood that $L_1$ players have a positive image of *ALLD* opponents ($7.2\%$ instead of 0%). This mismatch becomes even worse when errors and generosity act simultaneously (Fig. 2a,b, lower right panels). Now, 12.5% of $L_1$ players regard each other as bad, while they assign a good reputation to defectors in 13.9% of the cases. In particular, independent of whether or not there are errors, we observe that generosity undermines the accuracy of $L_1$ to assign correct reputations: (*i*) With and without errors, generosity reduces the likelihood that members of the $L_1$ subpopulation regard each other as good; (*ii*) With and without errors, generosity increases the likelihood that $L_1$ players assign a good reputation to defectors. We observe the same negative trend for all other leading eight norms (as another example, the case of $L_7$ is depicted in Fig. 2c,d).

To gain some intuition for these results, Fig. S1a considers a stylized example with three $L_1$ players and one *ALLD* player. Due to assessment generosity, some $L_1$ players may assign a good reputation to the defector (Fig. S1a, second panel). This can have two negative consequences for the relative performance of $L_1$: (*i*) Generous players provide help to the defector (Fig. S1a, second and third panel); (*ii*) They assign bad reputations to fellow $L_1$ players who do not show the same generosity (Fig. S1a, fourth panel). In this way, uncoordinated generosity can itself act as a seed of disagreement between generally cooperative players. Both of these effects of assessment generosity seem to undermine the robustness of the leading-eight norms in mixed populations, rather than enhancing it.

### Reputation dynamics under action generosity

**Figure 3:**
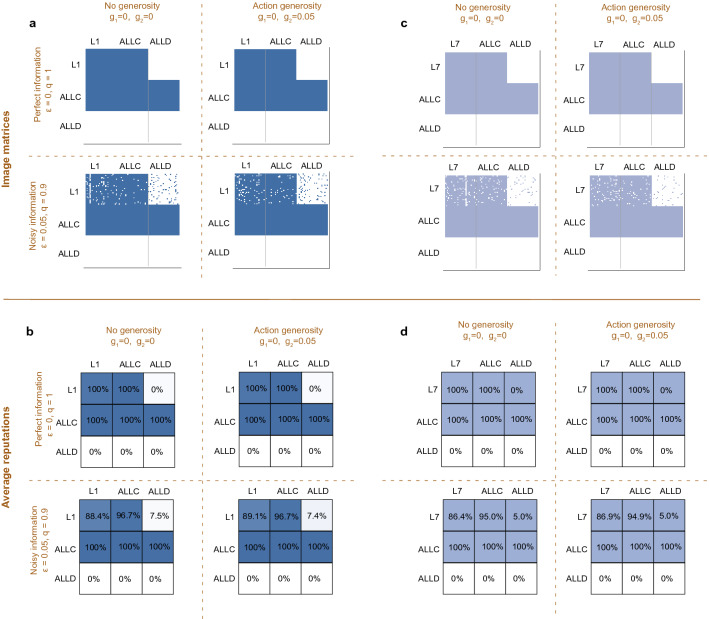
The effect of action generosity on the reputation dynamics. We consider the same basic setup as in Fig. 2, except that leading eight players now use action generosity instead of assessment generosity. (**a,b**) When the population consists of $L_1$, *ALLC*, and *ALLD*, action generosity results in more appropriate judgments. In the presence of errors, generosity makes $L_1$ players more likely to perceive each other as good (89.1% instead of 88.4%). Similarly, it makes them slightly less likely to perceive defectors as good (7.4% instead of 7.5%). Importantly, however, $L_1$ players with action generosity occasionally cooperate with *ALLD* opponents even if they regard them as bad. (**c**,**d**) We observe the same patterns for other leading eight norms. Here we again depict the case of $L_7$. Parameters are the same as in Fig. 2.

In a similar way, we can also explore the isolated effects of action generosity. To this end, we again consider populations that consist in equal proportions of $L_1$, *ALLC*, and *ALLD*, and we compare the outcomes with and without errors, and with and without action generosity (Fig. 3a). In contrast to assessment generosity, we observe that action generosity does not compromise the perceptions that $L_1$ players have of each other. In the absence of errors, all $L_1$ players regard each other as good, with or without action generosity (Fig. 3b, upper two panels). Once there are perception errors, action generosity can even improve $L_1$’s self-perception. Instead of regarding each other as bad with an average probability of 11.6% (in the case of errors but no generosity), $L_1$ players assign each other a bad reputation in 10.9% of the cases (with action generosity). Similarly, the chance to misperceive *ALLD* players as good is slightly diminished. $L_1$ players regard $7.5\%$ of their *ALLD* opponents as good without generosity, compared to $7.4\%$ with generosity (note, however, that under action generosity, an $L_1$ player may cooperate with *ALLD* players even when they have a bad reputation).

To gain some intuition for the differences between assessment generosity and action generosity, we revisit our previous stylized example with three $L_1$ players and one *ALLD* player (Fig. S1b). Similar to assessment generosity, we again observe that action generosity may lead $L_1$ players to cooperate with defectors (Fig. S1b, second and third panel). However, action generosity no longer seeds additional disagreements among the $L_1$ players (Fig. S1a, fourth panel). The main difference between assessment and action generosity is that instances of action generosity become common knowledge. Cooperating with a defector is a decision that others can see, whereas assigning a good reputation to a defector is a private decision which can lead to future misunderstandings.

These results suggest that with respect to the robustness of $L_1$, action generosity results in a trade-off. On the one hand, action generosity makes it more likely that $L_1$ players cooperate among each other when information is noisy. On the other hand, however, action generosity also increases the chance that help is given to undeserving defectors. Similar trade-offs can also be observed for other leading-eight norms (Fig. 3c,d shows the example of $L_7$). Whether the overall effect of this trade-off is favorable to the robustness of indirect reciprocity depends on the overall composition of the population. In populations in which leading eight players are abundant, the positive effects of increased forgiveness may outweigh the negative effects of providing help to free riders. In an evolutionary context, this composition of the population can change over time, depending on the relative success of each social norm. How generosity affects this evolutionary dynamics is what we explore in the following.

### Evolutionary dynamics under assessment and action generosity

While we have assumed in the previous sections that the population members use fixed social norms, here we consider how the abundance of different social norms may change over time. We assume that the evolution of social norms happens on a separate, much larger timescale than the updating of reputations. As a result, we may assume that by the time social norms change, the players’ reputations have reached a stationary state (as depicted by the average image matrices in Fig. 2b,d and 3b,d). Given these average images, we can compute the cooperation rate ${\hat{x}}_{ij}$ with which a player *i* provides help to player *j*. Using these cooperation rates, we compute player *i*’s average payoff as $\pi _i\!=\! \frac{1}{N\!-\!1} \sum _{j\ne i} b {\hat{x}}_{ji} - c {\hat{x}}_{ij}$. If a social norm allows a player to have a relatively high payoff, such a social norm is more likely to spread in a population on an evolutionary timescale.

To describe how successful social norms spread in a population, we consider a simple imitation dynamics based on pairwise comparison^45,46^. In every time step of the evolutionary process, some player *i* is randomly selected from the population to revise their social norm. With probability $\mu$, player *i* picks a new norm uniformly at random. With probability $1-\mu$, he instead chooses a role model *j* randomly from the remaining population. In that case, player *i* adopts *j*’s social norm with a probability given by the Fermi function^47–49^, $P(\pi _i,\pi _j)\!=\!(1\!+\!\exp [-s(\pi _j\!-\!\pi _i)])^{-1}$. Here, the parameter $s\!\ge \!0$ describes the strength of selection, which measures how relevant payoffs are for updating the norms. For $s\!=\!0$, updating happens at random. For increasing *s*, social norms with higher payoffs are increasingly likely to be imitated. The resulting stochastic process is ergodic, and gives rise to a stationary distribution, called selection-mutation equilibrium. The average cooperation rate or payoff in the population can be computed from there by weighting the payoffs of the individual norms with their abundance in equilibrium. In the following, we assume mutations are sufficiently rare, such that populations are homogenous most of the time^50–52^. Only occasionally, a mutant social norm arises. This mutant norm is then either adopted by the entire population or it goes extinct before the next mutant appears. To furthermore keep the system as simple as possible, we study the evolutionary competition between three social norms only: players can choose between *ALLC*, *ALLD*, and one of the leading eight social norms (similar to what we studied in the previous sections and again similar to much of the previous work in the field^20–23,38,53,54^). For all details, see “Methods”.

**Figure 4:**
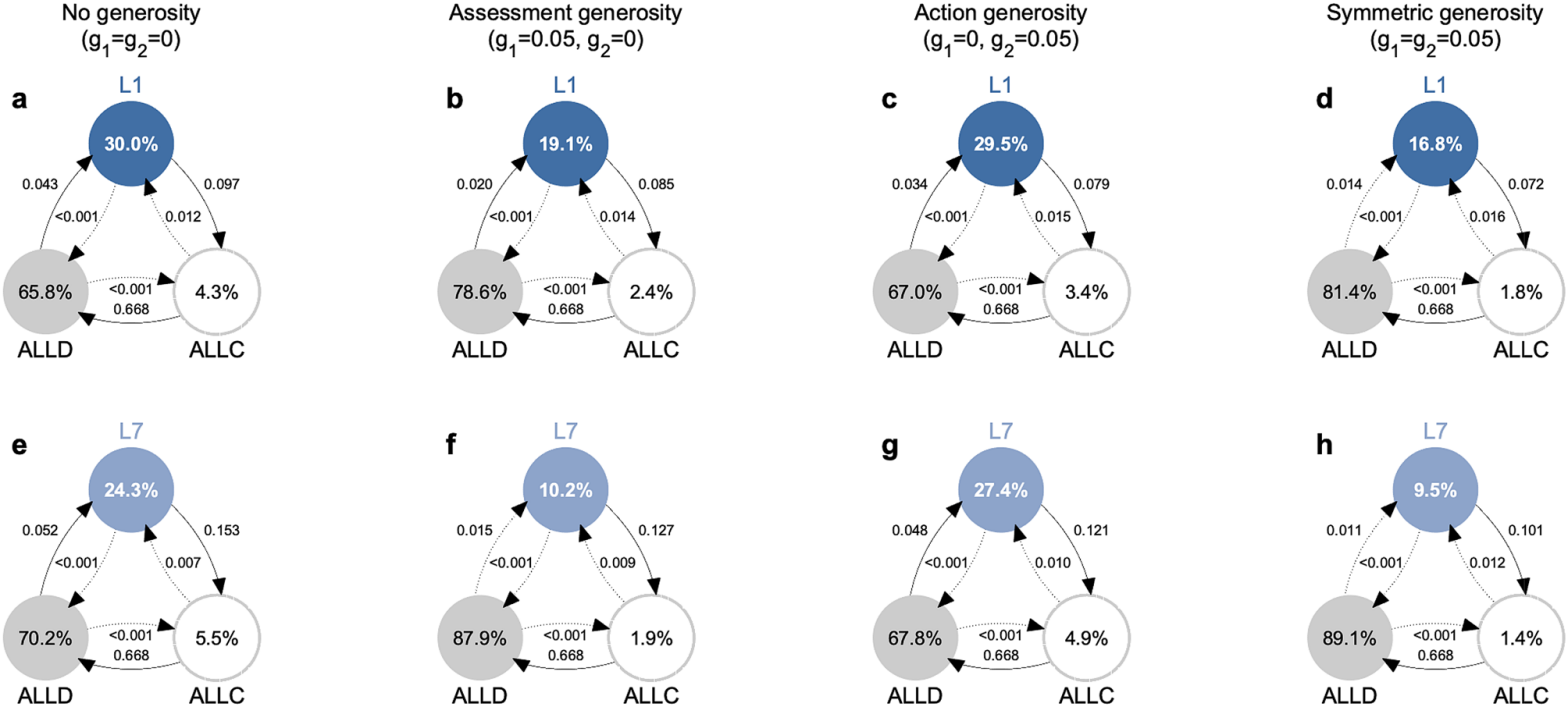
Evolution of the leading eight under assessment and action generosity. We simulate the evolutionary dynamics when players can choose among three different norms, a leading eight norm, *ALLC*, and *ALLD*. Social norms spread in the population according to a pairwise comparison process^45^, such that norms of players with high payoffs are more likely to spread. Here we depict results for the limit of rare mutations, such that populations are homogeneous most of the time^50–52^. Numbers in circles show how often each social norm is adopted on average. Arrows indicate how likely other social norms can invade a given resident population. Solid arrows indicate that the respective transition is more likely to occur than expected under neutrality, whereas dotted arrows indicate that the respective transition is comparably unlikely. We consider four scenarios, depending on whether leading eight players exhibit no generosity, only assessment generosity, only action generosity, or both variants of generosity. (**a**–**d**) The norm $L_1$ is most abundant without any generosity. (**e**–**h**) The norm $L_7$ is most abundant with action generosity. However, even in that case, it is played in less than 30% of time. Parameters: $N\!=\!50$, $\varepsilon \!=\!0.05$, $b\!=\!5$, $c\!=\!1$, $q\!=\!0.9$, using a strength of selection of $s\!=\!1$.

Figure 4a shows the resulting dynamics for the leading eight norm $L_1$ in the baseline case without any generosity^36^. Overall, $L_1$ is only played in 30% of the cases, as compared to 4.3% for *ALLC*, and 65.8% for *ALLD*. While $L_1$ is relatively robust against direct invasion by ${ALLD }$, it is vulnerable to indirect invasion by *ALLC*. Overall, the dynamics is similar to a rock-paper-scissors cycle: *ALLC* mutants are favored to invade into $L_1$, *ALLD* is favored to invade into *ALLC*, and $L_1$ can invade *ALLD* in turn. However, due to the relative robustness of *ALLD*, players only cooperate in a minority of cases. Adding a moderate amount of either assessment generosity (Fig. 4b) or action generosity (Fig. 4c) leaves the qualitative dynamics unchanged. However, both kinds of generosity make $L_1$ slightly more robust against invasion by *ALLC*, but less likely to invade into *ALLD*, as one may expect. The overall effect is slightly negative in the case of action generosity (the share of defectors increases from 65.8% to 67.0%), and substantially negative in the case of assessment generosity (where the share of defectors increases to 78.6%). For the given parameter values, we thus conclude that neither form of generosity favors the evolution of the social norm $L_1$.

This conclusion, however, can change for other leading eight social norms. To illustrate this point, Fig. 4e–h shows the resulting dynamics when *ALLC* and *ALLD* compete with the alternative norm $L_7$. The overall dynamics is similar to the case of $L_1$, with evolution leading in a rock-paper-scissors cycle from $L_7$ to *ALLC* to *ALLD* and back to $L_7$. Also the impact of generosity is similar. On the one hand, it reduces the risk of $L_7$ with respect to indirect invasions by *ALLC*; but on the other hand, it also reduces $L_7$’s ability to invade into *ALLD*. However, for $L_7$ we observe that the net result of these two opposing effects can be positive. In the case of action generosity, the share of $L_7$ increases to 27.4% (compared to 24.3% in the baseline scenario without any generosity). We note however that even in this case, in which generosity is favorable, the population is still most likely to settle at unconditional defection.

**Figure 5:**
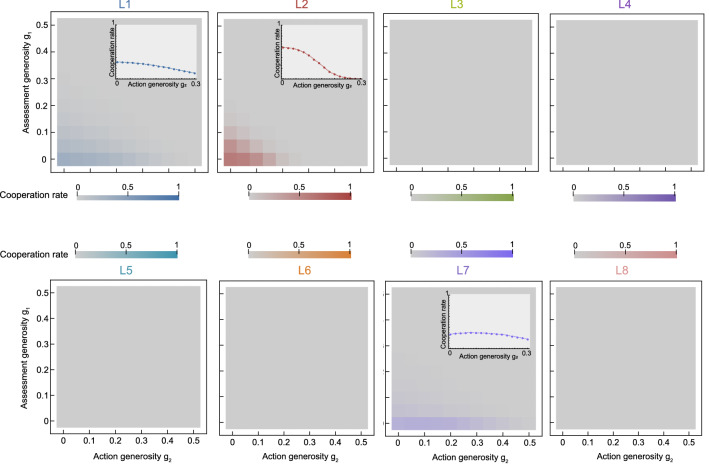
A systematic analysis of the effect of generosity on cooperation. For this figure, we repeat the evolutionary simulations shown in Fig. 4 for all leading eight norms. To explore the impact of generosity, we systematically vary how likely leading eight players exhibit assessment generosity ($g_1$, *y*-axis) and how likely the exhibit action generosity ($g_2$, *x*-axis). The color indicates the average cooperation rate of the population, according to the selection-mutation equilibrium of the evolutionary process (see “Methods”). In particular, grey indicates the absence of cooperation. We find that the five social norms $L_3-L_6$ and $L_8$, which fail to evolve in the baseline deterministic model ($g_1\!=\!g_2\!=\!0$), do not evolve in any generous form either, no matter in which combination. The norms $L_1$ and $L_2$ occasionally evolve, but they do not benefit from either form of generosity. Here, the achieved cooperation rate has its maximum in the origin. Only for $L_7$, the cooperation rate becomes maximal for a positive amount of action generosity, with $g_2\!\approx \!0.08$. The inserts show the cooperation rate as a function of $g_2$ for $g_1\!=\!0$. Parameters are the same as in Fig. 4.

To explore how robust these patterns are, we have repeated these simulations for all other leading eight social norms, and we have systematically varied how likely the leading eight are to engage in either assessment or action generosity (Fig. 5). These simulations exhibit the following regularities: (*i*) In the presence of noise, almost all of the leading eight norms have problems to evolve, irrespective of how generous they are. The only social norm that is able to reach an abundance of more than 50% is $L_2$ (called ‘consistent standing’^36^). However, this social norm is most successful without generosity, when $g_1\!=\!g_2\!=0$. (*ii*) In those cases in which the leading eight norm is played by a sizable fraction of the population ($L_1$, $L_2$, $L_7$), assessment generosity has a systematically negative effect on overall cooperation. In contrast, action generosity can promote cooperation, but only in the case of $L_7$, for which the optimal degree of generosity is $g_2\!\approx \!8\%$. However, even in that case, cooperation rates remain comparably low. For all other leading eight norms ($L_3$-$L_6$, $L_8$), cooperation fails to evolve altogether.

**Figure 6:**
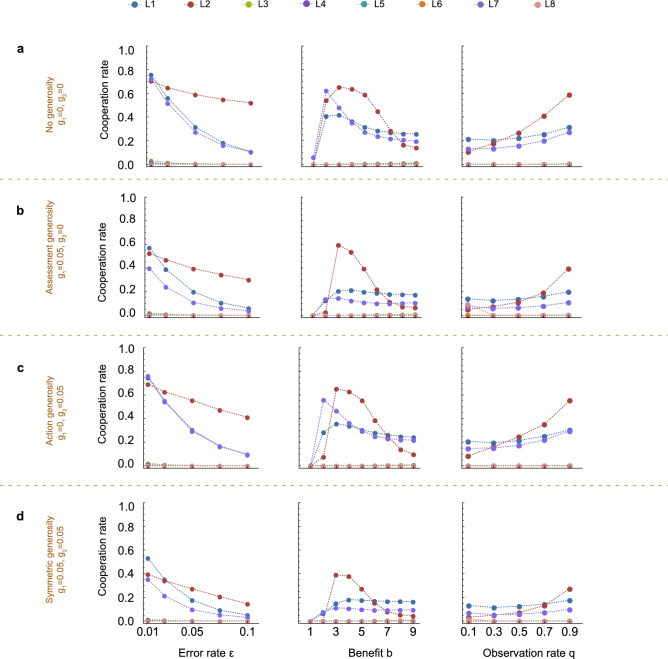
Generosity does not enhance the evolution of cooperation even when we vary parameter values. We vary the noise on observations $\varepsilon$, the benefit-to-cost ratio *b*/*c*, with $c=1$, and observation probability *q*. All other parameters remain constant at the values of Fig. 2. In each scenario, we plot the average cooperation rate of each individual leading eight norm when they compete against ALLD and ALLC. This again enables us to compare the generous $L_i$ (**b**–**d**) in three variants with their baseline counterparts (**a**). (**b**) For assessment generosity only, we find that compared to the baseline, the cooperation rate of the leading eight with generosity is reduced for all values of $\varepsilon$, *b*/*c* and *q*. The qualitative shape of the curves remains the same as in the case of no generosity. (**c**) In the case of action generosity only, the negative effect on cooperation rates is not as large as with assessment generosity. Yet, action generosity still fails to enhance cooperation, and is also detrimental for some parameter ranges, especially higher values of $\varepsilon$ and *b*/*c*. (**d**) For the leading eight with both kinds of generosity, cooperation rates are lowest across all parameter ranges and scenarios. Again, the shape of the curve is identical to its deterministic baseline in (**a**), but cooperation cannot evolve in the same way as in the baseline, where it is already limited.

We observe similar results as we vary the error rate $\varepsilon$, the benefit *b* of cooperation, and the observation rate *q* (Fig. 6). In all cases, cooperation is unlikely to evolve. Moreover, generosity seems to harm the evolution of cooperation rather than enhancing it (Fig. S2), despite its positive effect in homogeneous populations (Fig. S3).

## Discussion

Indirect reciprocity is a mechanism for cooperation that translates the principle of direct reciprocity to a population level^11–14^. When individuals use direct reciprocity, they ask how cooperative others have been in direct encounters. In contrast, when individuals use indirect reciprocity, they ask how others have behaved in general, in interactions with third parties. Once individuals take into account third party interactions, they also need to develop a sense of which behaviors should be regarded as bad, and which consequences bad reputations should have. In this way, indirect reciprocity has become the subfield of evolutionary game theory that explores the evolution of moral behavior^1^. To explore these questions, Ohtsuki and Iwasa^25,26^ have suggested eight social norms that can maintain cooperation. Their work, however, rests on the assumption that reputations are assigned publicly. In their framework it is a central authority that takes note of everyone’s behavior, and that assigns a good or a bad reputation in turn. In contrast, when population members make their own judgments and when information is noisy, Ohtsuki and Iwasa’s leading eight become less effective in sustaining cooperation^33–36^. Noise can introduce initial disagreements, such that different individuals assign different reputations to a given group member. Such disagreements can proliferate, because they also affect how future interactions of that group member are interpreted. As a result, minor initial disagreements can sometimes lead to a complete separation of the population. In such cases, the population fragments into distinct subpopulations that assign good reputations within their community and bad reputations to all outgroup members^55^.

Here, we have explored whether the leading eight social norms can be made more robust if they are equipped with an element of generosity. We distinguish two ways of being generous. When individuals engage in assessment generosity, they are more likely to assign a good reputation to someone they would usually regard as bad. When individuals engage in action generosity, they are more likely to cooperate with someone against whom they would usually defect. Although these two manifestations of generosity may follow a similar motive, our results suggest that they have different consequences. Assessment generosity leads to a private reassessment of somebody’s reputation. Unless this reassessment is mutually agreed upon, it can seed additional disagreements within a population. These disagreements can further undermine the robustness of the leading eight norms. In contrast, action generosity leads to a public display of cooperative behavior. While such generous acts may be misguided towards undeserving free riders, they do not generate the mutual disagreements that assessment generosity is susceptible to. As a result, we find that the effect of assessment generosity on cooperation is always negative, whereas action generosity can sometimes enhance cooperation. But even then, the leading eight norms have problems to sustain full cooperation in noisy environments.

We note here that the case of fully private, individual observations and reputation tracking constitutes an extreme case just as much as fully public information. We assume that for a more realistic model, the truth lies somewhere in the middle^56^. For example, gossip and communication help offset and mediate disagreements. Still, for a full understanding of the dynamics of indirect reciprocity, it should be useful to first explore the extreme cases. If not for practical reasons, these cases are still relevant because of their theoretical merit as boundary cases.

The failure of generosity to enhance cooperation may seem surprising. After all, previous work on direct reciprocity has shown that generosity is a powerful mechanism to reestablish mutual cooperation in the presence of noise^39–43^. Also among first-order social norms of indirect reciprocity, generosity can help to stabilize cooperation^44^. Our study can shed light on why generosity is conducive to cooperation in those cases, while it is detrimental in the case of the leading eight norms. For the leading eight norms, generosity entails a two-fold cost. The first cost is the immediate cost *c* that comes with any cooperative act. The second cost is the risk of losing one’s reputation by providing help to someone others regard as bad. In contrast, Generous Tit-for-Tat and Generous Scoring do not suffer from such a two-fold cost. Although the direct act of cooperating with someone is still costly, cooperators always obtain a good reputation. The optimal level of generosity in this case can be calculated by requiring that the positive reputation effect matches the negative immediate cost of cooperation^44^.

To model the impact of generosity, we make a number of simplifying assumptions. For one, we have considered a well-mixed population. While the assumption of well-mixed populations is common in the indirect reciprocity literature^11–13,19,25,26,36^ interesting effects may arise once a population’s network structure is taken into account. In the context of indirect reciprocity, this population structure may not only determine with whom players interact with, but also whose interactions they are able to observe. While population structure can enhance the evolution of cooperation in general^57–60^, it remains to be shown how it affects the dynamics of indirect reciprocity with and without generosity.

In addition, our analysis is based on computer simulations of the dynamics in finite populations. Instead, recent work has shown that analytical results are feasible in infinite populations if the players’ observations can be assumed to be independent^38,61^. While the dynamics in finite populations is perhaps more relevant for applications, an analytical treatment of generosity is another potential venue for future research.

Furthermore, we have assumed an “aligned” version of forgiveness: players have a single probability value each for generosity in assessments ($g_1$) and actions ($g_2$), respectively. They do not differentiate between cases within one component of their social norm, i.e. do not apply less stochasticity in scenarios they deem less “forgivable”. Note that a non aligned version of generosity with different probability of forgiveness for different observations or actions would certainly be more realistic. For example, it might well be that cooperation with a person deemed as bad should be subject to forgiveness more often than defection against a good person. However, this also intuitively demands a higher cognitive capacity from the players, who then need to prioritize some assessments and actions over others, and need to decide what they think are unforgivable offenses. That is, they need a more involved sense of morality. This points to a direction of interesting future work, which can take into account the importance that players assign to different scenarios they might encounter rather than applying an element of randomness uniformly to their actions and assessments. Exploring such “non-aligned” generosity component-wise would aid our understanding of which aspects of norms need to truly be set in stone, and which aspects deserve more flexibility in assessment and action. Our study with its negative result thus is only a first step towards understanding the effect of generosity for social norms.

Finally, previous work has shown that in more complex social situations, apologies are a necessary first step before forgiveness can occur. Without them, individuals lack discernment of what constitutes a justified or unjustified defection^62,63^. This makes our results intuitive from a sociology perspective: without a person being able to observe the intent behind an action, forgiveness does not help in re-establishing cooperation. It thus would potentially be worthwhile to explore the use of apology strategies in indirect reciprocity, similar to^63^, which analyzes direct reciprocity dynamics involving apology and forgiveness. Our study thus further serves to highlight the importance of shared information and coordination in a society when more complex social norms are applied. Simply acting in good faith every once in a while cannot balance out the intricate dynamics that result from disagreements in a population.

## Methods

### Reputation dynamics

We first describe how the reputations of players change over time. To this end, we assume social norms to be fixed for each individual. We record the reputations that players assign to one another in one image matrix *M*(*t*), which is updated in every step of the dynamics. An entry $m_{ij}=1$ ($m_{ij}=0$) means that player *i* regards player *j* as good (bad), respectively. In the following, we always start with all entries $m_{ij}=1$, i.e. in a state where everyone assigns a good reputation to everyone else, no matter the social norm. In every round, a donor and recipient are chosen at random, and the donor can choose whether to confer a benefit *b* to the recipient at own cost *c*. Her action is dependent on her norm as well as the reputation she assigns to herself and the recipient. This action is observed by her co-players with individual probability *q*. This observation is subject to misperception with probability $\varepsilon$. The players who observe the interaction then update the donor’s reputation according to their assessment rules. These updates are recorded in the matrix $M(t+1)$. We can iterate this process over many rounds and calculate the average image players have of each other. Specifically, if players have interacted for *T* rounds in total, the average image that player *i*’s assigns to player *j* is defined as $\frac{1}{T}\sum _{t=1}^T m_{ij}(t)$. The resulting average cooperation frequency ${\hat{x}}_{ij}$ enables us to calculate players’ payoffs for a fixed social norm as

1 $$\begin{aligned} \pi _i\!=\! \frac{1}{N\!-\!1} \sum _{j\ne i} b {\hat{x}}_{ji} - c {\hat{x}}_{ij}. \end{aligned}$$

We illustrate our approach in Figs. 2 and 3. In both cases, the upper panels (a,c) show snapshots of the image matrix at time $T=2 \times 10^6$. The lower panels (b,d) show the respective average images. In Fig. S3, we further present the results of reputation dynamics in a homogeneous population of leading eight players, by showing the average cooperation rate of each leading eight norm against itself.

We want to point out that in general, “good” and “bad” are merely labels with no inherent meanings. In particular, for each of the leading eight strategies, one could also define “mirror strategies”. According to these mirror strategies, acceptable behavior would be rewarded with a “bad” reputation, and players would be more likely to cooperate with “bad” individuals. If we are also to allow for such mirror strategies, it might be less clear how “assessment generosity” should be defined. After all, for the mirrors, assessment generosity should imply that forgiveness makes it more likely that players now assign a “bad” reputation to the respective player. However, in this study, we are only concerned with the leading eight strategies as defined by Ohtsuki and Iwasa^25^. In their definition, the terms “good” and “bad” already have some intrinsic meaning. Here, “good” is considered to mean the reputation label that, according to the leading eight’s action rule, makes it more likely for the respective player to receive cooperation. In our model, players thus unambiguously prefer to be labeled as “good”. In that case, assessment generosity is straightforward to define. It means that players are more likely to assign good reputation to their peers.

As one of our results, we show that action generosity may be more conducive to the evolution of cooperation than assessment generosity. This may appear unsurprising, because action generosity immediately affects cooperation. In case of action generosity, each instance of generosity directly translates into an act of cooperation. In contrast, in the case of assessment generosity, each instance of generosity only affects the respective player’s reputation. Whether this improved reputation translates into more received cooperation depends on the player’s further interactions. However, for many of the presented results, we actually do not explore the impact of generosity on the eventual cooperation rate. Rather, we explore how easily generosity can induce disagreements between leading eight players (Figs. 2 and 3), or how it affects the evolution of leading eight strategies (Fig. 4). Already on this level, we observe that action generosity is more favorable than assessment generosity. Furthermore, we show that even if action generosity by itself increases cooperation, we find that in most cases it actually has a negative effect on cooperation.

We also emphasize that our analysis technique is not limited to the restricted setup where only three social norms compete. The approach can be naturally extended by allowing population members to choose among additional norms. However, computational complexity increases rapidly in the number of considered social norms. Also, our results show that already when competing with only these two very simple other strategies, the leading eight norms are unstable. Thus, adding further social norms is unlikely to increase the stability of the leading eight norms and similarly unlikely to affect our conclusions.

### Evolutionary dynamics

In a next step, we explore which social norms the individuals themselves choose to adopt when their norms are no longer fixed. To describe this process formally, we assume players change their norms over a separate, longer time scale. On this longer time scale, we assume individuals adopt new social norms based on a pairwise comparison process^45^. According to this process, in every evolutionary time step, one player is randomly chosen from the population. With probability $\mu$, with $\mu$ the mutation rate, this player picks a new social norm at random from the respective set of available norms. In this work, players can choose from three social norms, a given leading eight norm, *ALLC*, and *ALLD*. Meanwhile, with $1-\mu$, the focal player randomly chooses a role model from the population. If the focal player’s payoff according to Eq. (1) is given by $\pi _i$ and the role model’s payoff is $\pi _j$, then the focal player adopts the role model’s norm with probability $P(\pi _i,\pi _j)\!=\!(1\!+\!\exp [-s(\pi _j\!-\!\pi _i)])^{-1}$. The parameter *s* is called the strength of selection. When *s* is small, imitation occurs largely at random. For larger *s*, however, players are most likely to imitate those role models with a higher payoff.

We note that in evolutionary game theory, imitation processes are often used as a standard model to describe the spread of strategies in a population. For this model to be sensible, it is necessary to assume however that players are able to infer their co-players’ strategies from their observed behaviors. This is straightforward for simple games, where strategies correspond to the action that a player chooses. It is less straightforward in games of indirect reciprocity. Here, individuals use more complex norms that are more difficult to learn by imitation. However, it may still be argued that players may be able to learn each other’s norms. For example, it is not unusual to assume that people discuss their world views and moral guidelines with others. This is what we implicitly assume for this study. Instead of imitation, one could also consider an alternative model by assuming that social norms spread through a birth-death process, i.e. that parents pass on their own social norms to their children. However, for the function we use to model imitation, the imitation process is equivalent to a birth-death process with exponential fitness mapping^46^. Thus, the results would not change.

This evolutionary process based on mutations and imitation is ergodic. Hence, it gives rise to a unique stationary distribution, which we refer to as the selection-mutation equilibrium. This equilibrium reflects how often each of the available social norms is adopted over time. In this work, we use the limit of rare mutations, which assumes that populations are homogeneous most of the time. When a mutation arises, it either fixes in the population or goes extinct before the next mutant appears. We can calculate this fixation probability of a mutant with social norm *M* into a resident population with social norm *R* explicitly as^64^

2 $$\begin{aligned} \rho _{MR}=\frac{1}{1+\sum _{i=1}^{n-1}\prod _{k=1}^{i} e^{-\beta \,(\pi _M(k)-\pi _R(k))}}. \end{aligned}$$

Here, $\pi _M(k)$ and $\pi _R(k)$ are the respective payoffs of mutants (*M*) and residents (*R*) when *k* individuals in the population employ the mutant norm. This means that we can describe the evolution of the social norms between three available norms in the rare mutation limit as a Markov chain with three states. These three states correspond to the respective homogeneous populations, i.e. all players using *ALLC*, all using *ALLD* or all players using a leading eight norm. Given the pairwise fixation probabilities according to Eq. 2, the respective transition matrix of this evolutionary Markov chain is given by

3 $$\begin{aligned} W=\left( \begin{array}{ccc} 1-\frac{1}{2}(\rho _{LC}+\rho _{LD}) &{}\frac{1}{2}\rho _{LC} &{}\frac{1}{2}\rho _{LD}\\ \frac{1}{2}\rho _{CL} &{}1-\frac{1}{2}(\rho _{CL}+\rho _{CD}) &{}\frac{1}{2}\rho _{CD}\\ \frac{1}{2}\rho _{DL} &{}\frac{1}{2}\rho _{DC} &{}1-\frac{1}{2}(\rho _{DL}+\rho _{DC}) \end{array} \right) . \end{aligned}$$

The stationary distribution of this transition matrix is the selection-mutation equilibrium of the process for rare mutations^50^. Given this equilibrium, we can compute how often players cooperate on average by taking the average cooperation rate of each homogeneous population, and multiplying it by how often we are to observe the respective homogeneous population in equilibrium.

We use this approach in Fig. 4–Fig. S2, where we first simulated the reputation dynamics for all possible population compositions, $(n_L,n_C,n_D)$, with $N=n_L+n_C+n_D = 50$ and $10^6$ steps each. Here, $n_L$ stands for the number of players using a (generous) leading eight norm, $n_C$ for the number of players using *ALLC*, and $n_D$ for the number of *ALLD* players. Payoffs are computed with Eq. 1, as explained in the subsection on the reputation dynamics.

Specific methods employed for the figures. Figure 2 shows the results of reputation dynamics in a population of $N\!=\!90$ players. The population composition is as follows: 1/3 uses *ALLD*, 1/3 uses *ALLC*, and 1/3 uses a leading eight norm. We consider the cases of $L_1$ (**a,b**) and $L_7$ (**c,d**). We additionally differentiate between four parameter setups. For these setups we first distinguish whether information is perfect or noisy (i.e., whether $\varepsilon =0$ and $q=1$, or $\varepsilon = 0.05$ and $q=0.9$). In addition, we distinguish whether or not players engage in assessment generosity (i.e., whether $g_1=0.05$ or $g_1=0$, while $g_2 = 0$ throughout).

Figure 3 uses exactly the same method as Fig. 2, but considers the case that players use action generosity ($g_1=0, g_2=0.05$) instead of assessment generosity.

In Fig. 4, we show the abundance of *ALLC*, *ALLD* and either $L_1$ or $L_7$ in the selection-mutation equilibrium. We compare a scenario without generosity ($g_1=g_2=0$) to scenarios with assessment generosity only ($g_1=0.05, g_2=0$), action generosity only ($g_1=0, g_2=0.05$), and symmetric generosity ($g_1=g_2=0.05$). Parameters are $b=5, c=1, s=1, \varepsilon =0.05, q=0.9$.

In Fig. 5, we systematically explore the effect of generosity on cooperation in the selection-mutation equilibrium for all leading eight norms. We use the same baseline parameters as in Fig. 4, while varying both assessment (*y*-axis) and action (*x*-axis) generosity in steps of 0.05 between 0 and 0.5. To visualize the impact of action generosity on the three norms $L_1$, $L_2$, and $L_7$ in more detail, we also ran more fine-grained simulations where we varied $g_2$ in steps of 0.01, while $g_1=0$. These results are presented in the inset panels **a, b, g**.

For Fig. 6, we again employed the same method as in Figs. 4 and 5. Here, we rerun the simulations for varying benefit *b* , noise $\varepsilon$ , and observation probability *q*. We again compare **a**, the baseline without generosity ($g_1=g_2=0$) to the three scenarios of (**b**), assessment generosity only ($g_1=0.05, g_2=0$), (**c**), action generosity only ($g_1=0, g_2=0.05$), and (**d**), both kinds of generosity ($g_1=g_2=0.05$). The other parameters remain the same as in the previous figures.

Figure S2 explores the effect of generosity in a perfect information scenario, for an error rate of $\varepsilon =0$ and an observation probability of $q=1$. We once more consider three scenarios: assessment generosity only with $g_1=0.05$ (**a**), action generosity only with $g_2 =0.05$ (**b**), and symmetric generosity, with $g_1=g_2=0.05$ (**c**). Methods employed are as in the previous evolutionary figures Fig. 4, 5, 6. All other parameters remain the same.

For Fig. S3, we vary generosity levels in all three scenarios (assessment generosity only, action generosity only, symmetric generosity) when considering reputation dynamics in a homogeneous population of leading eight players with noisy environment ($\varepsilon =0.05$, $q=0.9$). We plot the average cooperation rate of each employed leading eight norm against itself.

## Supplementary information

Supplementary Information 1.

## Data Availability

The raw data generated for the main text is available from the authors upon request. All simulations and numerical calculations have been performed with Python 2.7. The Python script used to simulate the reputation dynamics and calculate the selection-mutation equilibrium as well as average cooperation rates is available online at https://osf.io/hw6ay/?view_only=3802aa5e3a0f400b90cc004a461f999e!.

## References

[1] Alexander R (1987). The Biology of Moral Systems.

[2] Jacquet J, Hauert C, Traulsen A, Milinski M (2011). Shame and honour drive cooperation. Biol. Let..

[3] Fehr E (2004). Don’t lose your reputation. Nature.

[4] Bolton GE, Katok E, Ockenfels A (2005). Cooperation among strangers with limited information about reputation. J. Public Econ..

[5] Wedekind C, Braithwaite VA (2002). The long-term benefits of human generosity in indirect reciprocity. Curr. Biol..

[6] Sugden R (1986). The Economics of Rights, Co-operation and Welfare.

[7] Semmann D, Krambeck HJ, Milinski M (2004). Strategic investment in reputation. Behav. Ecol. Sociobiol..

[8] Kandori M (1992). Social norms and community enforcement. Rev. Econ. Stud..

[9] Milinski M, Semmann D, Krambeck HJ (2002). Reputation helps solve the ‘tragedy of the commons’. Nature.

[10] Cuesta JA, Gracia-Lázaro C, Ferrer A, Moreno Y, Sánchez A (2015). Reputation drives cooperative behaviour and network formation in human groups. Sci. Rep..

[11] Nowak MA, Sigmund K (2005). Evolution of indirect reciprocity. Nature.

[12] Sigmund K (2010). The Calculus of Selfishness.

[13] Sigmund K (2012). Moral assessment in indirect reciprocity. J. Theor. Biol..

[14] Okada I (2020). A review of theoretical studies on indirect reciprocity. Games.

[15] Axelrod R, Hamilton WD (1981). The evolution of cooperation. Science.

[16] Hilbe C, Chatterjee K, Nowak MA (2018). Partners and rivals in direct reciprocity. Nat. Hum. Behav..

[17] García J, van Veelen M (2018). No strategy can win in the repeated prisoner’s dilemma: Linking game theory and computer simulations. Front. Robot. AI.

[18] Glynatsi NE, Knight VA (2021). A bibliometric study of research topics, collaboration and centrality in the field of the Iterated Prisoner’s Dilemma. Human. Soc. Sci. Commun..

[19] Nowak MA, Sigmund K (1998). Evolution of indirect reciprocity by image scoring. Nature.

[20] Nowak MA, Sigmund K (1998). The dynamics of indirect reciprocity. J. Theor. Biol..

[21] Berger U (2011). Learning to cooperate via indirect reciprocity. Games Econom. Behav..

[22] Panchanathan K, Boyd R (2003). A tale of two defectors: The importance of standing for evolution of indirect reciprocity. J. Theor. Biol..

[23] Brandt H, Sigmund K (2004). The logic of reprobation: Assessment and action rules for indirect reciprocation. J. Theor. Biol..

[24] Leimar O, Hammerstein P (2001). Evolution of cooperation through indirect reciprocity. Proc. R. Soc. B.

[25] Ohtsuki H, Iwasa Y (2004). How should we define goodness?—Reputation dynamics in indirect reciprocity. J. Theor. Biol..

[26] Ohtsuki H, Iwasa Y (2006). The leading eight: Social norms that can maintain cooperation by indirect reciprocity. J. Theor. Biol..

[27] Pacheco JM, Santos FC, Chalub FACC (2006). Stern-judging: A simple, successful norm which promotes cooperation under indirect reciprocity. PLoS Comput. Biol..

[28] Brandt H, Sigmund K (2005). Indirect reciprocity, image scoring, and moral hazard. Proc. Nat. Acad. Sci. USA.

[29] Ohtsuki H, Iwasa Y, Nowak MA (2009). Indirect reciprocity provides only a narrow margin of efficiency for costly punishment. Nature.

[30] Sasaki T, Okada I, Nakai Y (2017). The evolution of conditional moral assessment in indirect reciprocity. Sci. Rep..

[31] Santos FP, Santos FC, Pacheco JM (2018). Social norm complexity and past reputations in the evolution of cooperation. Nature.

[32] Xu, J., García, J. & Handfield T. Cooperation with bottom-up reputation dynamics. AAMAS’19 (2019).

[33] Uchida S (2010). Effect of private information on indirect reciprocity. Phys. Rev. E.

[34] Uchida S, Sasaki T (2013). Effect of assessment error and private information on stern-judging in indirect reciprocity. Chaos Solitons Fractals.

[35] Okada I, Sasaki T, Nakai Y (2017). Tolerant indirect reciprocity can boost social welfare through solidarity with unconditional cooperators in private monitoring. Sci. Rep..

[36] Hilbe C, Schmid L, Tkadlec J, Chatterjee K, Nowak MA (2018). Indirect reciprocity with private, noisy, and incomplete information. Proc. Natl. Acad. Sci. USA.

[37] Krellner, M. & Han, T. A. Pleasing enhances indirect reciprocity under private assessment. *Artif. Life.* (2021). 10.1038/s41598-021-02677-2 PMC8654852 34880264

[38] Radzvilavicius AL, Stewart AJ, Plotkin JB (2019). Evolution of empathetic moral evaluation. eLife..

[39] Molander P (1985). The optimal level of generosity in a selfish, uncertain environment. J. Conflict Resolut..

[40] Nowak MA, Sigmund K (1992). Tit for tat in heterogeneous populations. Nature.

[41] Martinez-Vaquero LA, Cuesta JA, Sanchez A (2012). Generosity pays in the presence of direct reciprocity: A comprehensive study of 2x2 repeated games. PLoS ONE.

[42] Stewart AJ, Plotkin JB (2013). From extortion to generosity, evolution in the Iterated Prisoner’s Dilemma. Proc. Nat. Acad. Sci. USA.

[43] Hilbe C, Wu B, Traulsen A, Nowak MA (2014). Cooperation and control in multiplayer social dilemmas. Proc. Nat. Acad. Sci. USA.

[44] Schmid, L., Chatterjee, K., Hilbe, C. & Nowak, M. A. A unified framework of direct and indirect reciprocity. *Nat. Hum. Behav.* (2021). 10.1038/s41562-021-01114-8 33986519

[45] Traulsen A, Pacheco JM, Nowak MA (2007). Pairwise comparison and selection temperature in evolutionary game dynamics. J. Theor. Biol..

[46] Wu B, Bauer B, Galla T, Traulsen A (2015). Fitness-based models and pairwise comparison models of evolutionary games are typically different–even in unstructured populations. New J. Phys..

[47] Blume LE (1993). The statistical mechanics of strategic interaction. Games Econom. Behav..

[48] Szabó G, Tőke C (1998). Evolutionary Prisoner’s Dilemma game on a square lattice. Phys. Rev. E.

[49] Traulsen A, Nowak MA, Pacheco JM (2006). Stochastic dynamics of invasion and fixation. Phys. Rev. E.

[50] Fudenberg D, Imhof LA (2006). Imitation processes with small mutations. J. Econ. Theory.

[51] Wu B, Gokhale CS, Wang L, Traulsen A (2012). How small are small mutation rates?. J. Math. Biol..

[52] McAvoy A (2015). Comment on "Imitation processes with small mutations". J Econ Theory.

[53] Brandt H, Sigmund K (2006). The good, the bad and the discriminator—Errors in direct and indirect reciprocity. J. Theor. Biol..

[54] Santos FP, Santos FC, Pacheco JM (2016). Social norms of cooperation in small-scale societies. PLoS Comput. Biol..

[55] Oishi K, Shimada T, Ito N (2013). Group formation through indirect reciprocity. Phys. Rev. E.

[56] Okada, I., Yamamoto, H. & Uchida, S. Hybrid assessment scheme based on the stern-judging rule for maintaining cooperation under indirect reciprocity. *Games.* **11**(1), (2020).

[57] Lieberman E, Hauert C, Nowak MA (2005). Evolutionary dynamics on graphs. Nature.

[58] Ohtsuki H, Nowak MA (2008). Evolutionary stability on graphs. J. Theor. Biol..

[59] Ohtsuki H, Nowak MA (2007). Direct reciprocity on graphs. J. Theor. Biol..

[60] Szabó G, Fáth G (2007). Evolutionary games on graphs. Phys. Rep..

[61] Okada I, Sasaki T, Nakai Y (2018). A solution for private assessment in indirect reciprocity using solitary observation. J. Theor. Biol..

[62] Han TA, Moniz Pereira L, Santos FC (2011). Intention recognition promotes the emergence of cooperation. Adapt. Behav..

[63] Martinez-Vaquero LA, Han TA, Pereira LM, Lenaerts T (2015). Apology and forgiveness evolve to resolve failures in cooperative agreements. Sci. Rep..

[64] Traulsen A, Hauert C, Schuster HG (2009). Stochastic evolutionary game dynamics. Reviews of Nonlinear Dynamics and Complexity.

