# Supplementary material for: The evolution of indirect reciprocity under action and assessment generosity
Source: Sci Rep. 2021 Aug 31;11:17443. doi: 10.1038/s41598-021-96932-1 (PMC8408181; doi:10.1038/s41598-021-96932-1)
Supplement: Supplementary file 1 — Supplementary Information 1. [file 41598_2021_96932_MOESM1_ESM.pdf]

Supplementary Information: The evolution of indirect reciprocity under action and assessment generosity

Laura Schmid[1,*], Pouya Shati[2], Christian Hilbe[3], Krishnendu Chatterjee[1]
[1]IST Austria, Am Campus 1, 3400 Klosterneuburg, Austria
[2]Department of Computer Science, University of Toronto, ON M5S, Canada
[3]Max Planck Research Group Dynamics of Social Behavior, Max Planck Institute for Evolutionary Biology, 24306 Ploen, Germany
[*]E-mail: lschmid@ist.ac.at

August 9, 2021

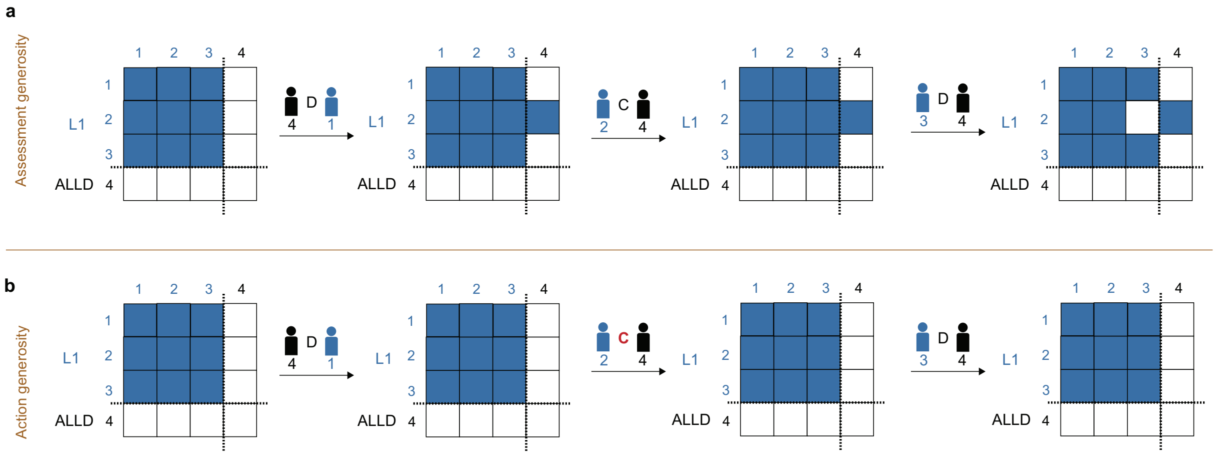

Figure S1: The mechanisms of assessment and action generosity differ. **a,** Assessment generosity g_1 works like an additional private error rate, and seeds disagreements that can later proliferate in a population using any generous leading eight norm, even when information is not noisy or incomplete. We show an example situation where such a disagreement arises in a population of 3 generous players and one ALLD player. When a generous player deems the ALLD player as good despite having observed or received a defection from her, he is setting up ALLD for higher reward and the generous social norm for in-fighting later on. **b,** Action generosity means more frequent cooperation with players deemed bad. This does not change the reputation dynamics for L1 or L7, as these two norms either never judge any cooperative action as bad or do not change their opinion about a cooperative player in the first place. However, action generosity still ends up giving benefits to defectors, who can then exploit the leading eight players' generosity. Intuitively, action generosity can be compared to a "public error" - this way it either actively harms in social norms where cooperation with bad players is judged as bad, or harms on the level of norm evolution.

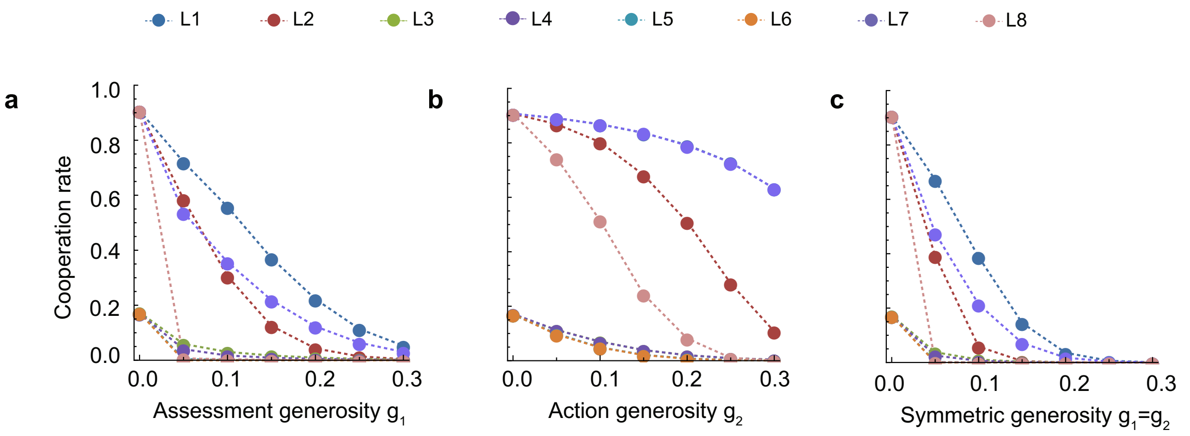

Figure S2: Generosity in itself inhibits the evolution of cooperation. We consider an error-free scenario ($\varepsilon = 0$) with perfect observation ($q = 1$), hereon called a "perfect information scenario", and calculate the cooperation rate in equilibrium as a function of generosity. **a,** In the case of assessment generosity only as well as when both variants of generosity are at play (**c**), there is a quick decline of cooperation for all generous leading eight as generosity increases. L1 does a bit better than the rest, but suffers the same losses once generosity is past 1%. **b,** When we consider only action generosity, the picture is slightly different: L1 and L7 can keep up a higher level of cooperation with a rate of around 85% until a probability of forgiveness at around 0.25. L2 also fares better in this setup than in the two other scenarios, but shows a decline in performance earlier on, at around $g_2 = 0.15$. It thus stands to reason that generosity in itself introduces disagreements and opportunities for ALLD to gain an advantage. Parameters: $N = 50, \varepsilon = 0, q = 1$. $b = 5$, $c = 1$, $s = 1$, in the limit of rare mutations $\mu \to 0$.

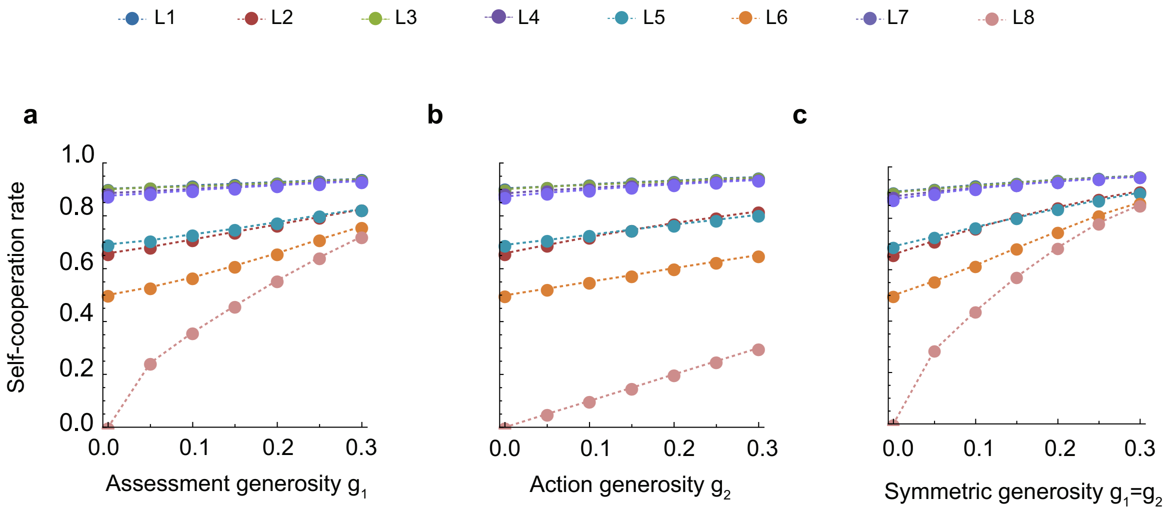

Figure S3: The self-cooperation rate in homogeneous populations of generous leading eight players benefits from generosity. **a-c,** We consider homogeneous populations consisting of $N = 50$ players using generous leading eight norms in a noisy environment, and calculate their norm's cooperation rate against itself. We find that as generosity increases, so does self-cooperation. This is true for assessment generosity only (**a**), action generosity only (**b**), as well as symmetric generosity ($g_1 = g_2$) (**c**). This result suggests that only when evolution of the social norms is at play and the generous norms have to compete against other norms, forgiveness is a hindrance. Single generous norms in isolation fare better in noisy environments than their deterministic variants. This is in line with the intuition that forgiveness helps balance out noise-related misinterpretations of cooperation as defection. Parameters: $N = 50, \varepsilon = 0.05, q = 0.9$.
